# DNA Methylation and Gene Expression of the Cysteinyl Leukotriene Receptors as a Prognostic and Metastatic Factor for Colorectal Cancer Patients

**DOI:** 10.3390/ijms24043409

**Published:** 2023-02-08

**Authors:** Souvik Ghatak, Shakti Ranjan Satapathy, Anita Sjölander

**Affiliations:** Cell and Experimental Pathology, Department of Translational Medicine, Lund University, 205 02 Malmö, Sweden

**Keywords:** *CYSLTR1*, *CYSLTR2*, methylation, prognosis, metastasis, colorectal cancer, EMT

## Abstract

Colorectal cancer (CRC), one of the leading causes of cancer-related deaths in the western world, is the third most common cancer for both men and women. As a heterogeneous disease, colon cancer (CC) is caused by both genetic and epigenetic changes. The prognosis for CRC is affected by a variety of features, including late diagnosis, lymph node and distant metastasis. The cysteinyl leukotrienes (CysLT), as leukotriene D_4_ and C_4_ (LTD_4_ and LTC_4_), are synthesized from arachidonic acid via the 5-lipoxygenase pathway, and play an important role in several types of diseases such as inflammation and cancer. Their effects are mediated via the two main G-protein-coupled receptors, CysLT_1_R and CysLT_2_R. Multiple studies from our group observed a significant increase in CysLT_1_R expression in the poor prognosis group, whereas CysLT_2_R expression was higher in the good prognosis group of CRC patients. Here, we systematically explored and established the role of the CysLTRs, cysteinyl leukotriene receptor 1(*CYSLTR1)* and cysteinyl leukotriene receptor 2 (*CYSLTR2)* gene expression and methylation in the progression and metastasis of CRC using three unique in silico cohorts and one clinical CRC cohort. Primary tumor tissues showed significant *CYSLTR1* upregulation compared with matched normal tissues, whereas it was the opposite for the *CYSLTR2*. Univariate Cox proportional-hazards (CoxPH) analysis yielded a high expression of *CYSLTR1* and accurately predicted high-risk patients in terms of overall survival (OS; hazard ratio (HR) = 1.87, *p* = 0.03) and disease-free survival [DFS] Hazard ratio [HR] = 1.54, *p* = 0.05). Hypomethylation of the *CYSLTR1* gene and hypermethylation of the *CYSLTR2* gene were found in CRC patients. The M values of the CpG probes for *CYSLTR1* are significantly lower in primary tumor and metastasis samples than in matched normal samples, but those for *CYSLTR2* are significantly higher. The differentially upregulated genes between tumor and metastatic samples were uniformly expressed in the high-*CYSLTR1* group. Two epithelial–mesenchymal transition (EMT) markers, E-cadherin (*CDH1*) and vimentin (*VIM*) were significantly downregulated and upregulated in the high-*CYSLTR1* group, respectively, but the result was opposite to that of *CYSLTR2* expression in CRC. *CDH1* expression was high in patients with less methylated *CYSLTR1* but low in those with more methylated *CYSLTR2*. The EMT-associated observations were also validated in CC SW620 cell-derived colonospheres, which showed decreased E-cadherin expression in the LTD_4_ stimulated cells, but not in the CysLT_1_R knockdown SW620 cells. The methylation profiles of the CpG probes for CysLTRs significantly predicted lymph node (area under the curve [AUC] = 0.76, *p* < 0.0001) and distant (AUC = 0.83, *p* < 0.0001) metastasis. Intriguingly, the CpG probes cg26848126 (HR = 1.51, *p* = 0.03) for *CYSLTR1*, and cg16299590 (HR = 2.14, *p* = 0.03) for *CYSLTR2* significantly predicted poor prognosis in terms of OS, whereas the CpG probe cg16886259 for *CYSLTR2* significantly predicts a poor prognosis group in terms of DFS (HR = 2.88, *p* = 0.03). The *CYSLTR1* and *CYSLTR2* gene expression and methylation results were successfully validated in a CC patient cohort. In this study, we have demonstrated that CysLTRs’ methylation and gene expression profile are associated with the progression, prognosis, and metastasis of CRC, which might be used for the assessment of high-risk CRC patients after validating the result in a larger CRC cohort.

## 1. Introduction

Colorectal cancer (CRC) is one of the leading causes of cancer-related deaths, ranking third for both men and women [1,2]. A decreasing trend in the metastatic CRC (mCRC) has been observed during the last years after the introduction of screening programs. However, the treatment strategies are still complicated due to the large number of patients detected with lymph node or distant metastasis. Metastasis is one of the most serious issues that reduce the survival of CRC patients and the effectiveness of their treatment. The median disease-free survival (DFS) and overall survival (OS) of patients with lymph node or distant mCRC have significantly increased because of late diagnosis or implementation of combined chemotherapy and drug treatment. Hence, there is a need to identify potentially related genes and their roles in the metastasis development. In addition, identifying robust predictive and prognostic markers to be validated in population-based cohorts could improve survival for mCRC patients.

Leukotrienes are inflammatory lipid mediators produced in different cells type from arachidonic acid via the 5-lipoxygenase pathway [3]. The two main cysteinyl leukotrienes (CysLTs) are LTC_4_, and LTD_4_, well known for their inflammatory effect caused by the CysLTs in cancer [3,4,5]. There has been an emerging role for CysLTs in cancer [6,7,8]. The roles of the two cysteinyl leukotriene receptors, CysLT_1_R and CysLT_2_R, have been well-reported for different types of cancer [6,7,8]. CysLT_1_R and CysLT_2_R are the high-affinity receptors for LTD_4_ and LTC_4_, respectively [4,5]. Montelukast and zafirlukast, CysLT_1_R antagonists, were found to possess dose-dependent chemopreventive effects against several cancers [9]. Moreover, CysLT_1_R overexpression has been observed in different types of cancers, including colorectal cancer, breast cancer, prostate cancer, urothelial transitional cell carcinoma and renal cell carcinoma [10,11,12,13,14,15,16,17]. The expression of CysLT_1_R and CysLT_2_R varies in various human tissues, including the respiratory and gastrointestinal systems and the brain [3,6,7,8,9]. The expression of CysLT_2_R, compared with that of CysLT_1_R, has been found to be higher in normal mucosa compared to its matched cancer tissues; however, CysLT_1_R expression was higher than CysLT_2_R expression in tumor samples [6]. A recurrent, hotspot mutation, p.Leu129Gln in *CYSLTR2*, an oncogene driver in uveal melanoma and leptomeningeal melanocytic tumors, leads to the activation of endogenous Gαq signaling and contributes to tumor progression in vivo [18,19]. To our knowledge, no reported evidence exists for genetic alterations of these receptors, resulting in good prognosis for CRC patients with low CysLT_1_R and high CysLT_2_R expressions.

A crucial step in cancer invasion and metastasis is the epithelial–mesenchymal transition (EMT). Cell adhesion molecules, such as E-cadherin, must be suppressed in order for EMT to occur. Furthermore, EMT results in the decline in E-cadherin and the increase in mesenchymal markers such as vimentin [20]. E-cadherin is decreased by LTD_4_ in CC cell lines [21]. Whereas MMP-9 is a metallopeptidase known to induce EMT in breast cancer cells, it is also induced by LTD_4_ in SW480 CC cells [22]. In a recent study, under basal conditions, *Cysltr1^−/−^* mice had higher expression of E-cadherin mRNA than wild-type mice [23]. Furthermore, in an earlier report from our group, a significant reduction in E-cadherin levels was observed in HCT-116 CC cells after LTD_4_ stimulation and GSK-3ß inhibition [21].

DNA methylation is a common and early epigenetic event that controls gene expression without changing genomic DNA sequences, and it consists of the attachment of methyl groups primarily to cytosines in the context of CpG dinucleotides. Multiple studies have proven that DNA methylation plays a key role in disease development by controlling driver gene expression [24], especially in cancers [25,26,27,28]. It is generally believed that the promoter regions of tumor suppressor genes (TSGs) are hypermethylated and repressed, whereas oncogenes are hypomethylated and abnormally active in cancer cells [29]. Furthermore, differentially methylated CpG probes could be used as biomarkers for the prognosis, metastasis prediction and treatment response of different cancers [25,30]. For patients presenting with a secondary tumor or metastases, DNA methylation markers can also be used to predict the origin of primary tumors [31]. Moreover, tissue- and disease-specific gene expression is also associated with DNA methylation differences in CpG islands [32]. Although several studies have investigated the DNA methylation profile of CRC [33], no sensitive or specific biomarkers have been identified for the prognosis of mCRC.

Building upon this evidence, we performed a systematic and comprehensive analysis of the methylation and expression of the *CYSLTR1* and *CYSLTR2* genes in the TCGA-COADREAD cohort, and the results were validated in two independent in silico cohorts (GSE77955 cohort from the GEO database and E-MTAB-8148 from the EMBL—EBI). We demonstrate the role of CysLT_1_R and CysLT_2_R in colorectal cancer progression and metastasis. Furthermore, we performed a comprehensive analysis of the methylation status of the *CYSLTR1* and *CYSLTR2* genes, which serve as drivers for CRC prognosis and metastasis development and could be used as prognostic markers for CRC patients.

## 2. Results

The Cancer Genome Atlas for colorectal cancer (TCGA-COADREAD) contains a gene expression profile from the Illumina HiSeq 2000 RNA sequencing platform and a DNA methylation profile from Illumina Infinium HumanMethylation450 platform with 416 primary colorectal cancer (COAD n = 284, READ n = 91) and matched normal (n = 41) samples (see Table 1 and study plan). Both the CysLT receptor (*CYSLTR1* and *CYSLTR2*) genes were expressed in the CRC samples, confirming their potential disease relevance. The differentially global methylated CpGs in TCGA-COADREAD dataset with δ|β|  >  0.25 and adjusted *p*  <  0.05 is visualized in Supplementary Figure S1. There were 16,122 hyper-methylated and 8736 hypomethylated CpGs observed in the TCGA-COADREAD dataset. Among the three CpG probes (cg00813999, cg10091155 and cg26848126) for the *CYSLTR1* gene, two CpG probes (cg00813999 and cg26848126) exhibited a methylation profile, whereas, among the five CpG probes (cg06038701, cg06322064, cg16299590, cg16886259 and cg18236297) for the *CYSLTR2* gene, two CpG probes (cg16299590 and cg16886259) exhibited a methylation profile for CRC.

**Figure 1:**
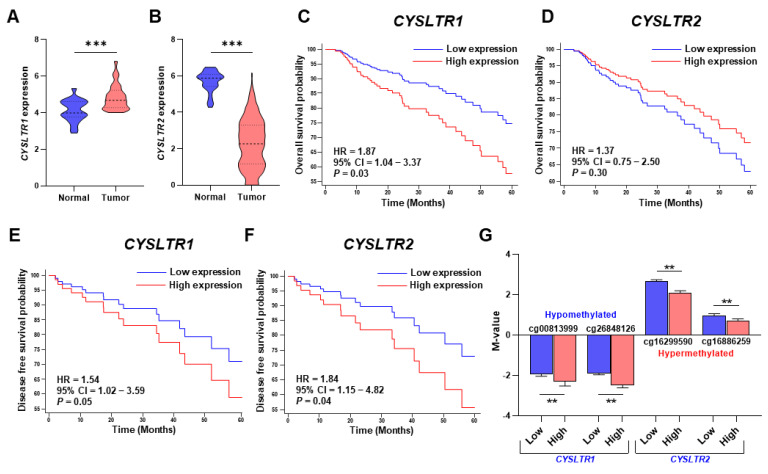
Violin plot of the relative expression and Kaplan–Meier survival plot of the *CYSLTR1* and *CYSLTR2* genes in the TCGA-COADREAD cohort. Relative expression of *CYSLTR1* (**A**) and *CYSLTR2* (**B**) between normal and tumor samples. Five-year Kaplan–Meier overall survival plots for *CYSLTR1* (**C**) and *CYSLTR2* (**D**). Five-year Kaplan–Meier disease-free survival plots for *CYSLTR1* (**E**) and *CYSLTR2* (**F**). (**G**) Correlation of M-values for CpG probes with *CYSLTR1* (cg00813999, cg26848126) and *CYSLTR2* (cg16299590, cg16886259) gene expression in tumor samples. ** *p* < 0.01; *** *p* < 0.001.

*CYSLTR1* and *CYSLTR2* expression were significantly differentially regulated between CRC tumor tissues and corresponding normal tissues in the TCGA-COADREAD cohort. *CYSLTR1* expression was significantly upregulated in CRC tumor tissues compared with matched normal tissues (*p* = 0.0004, paired t-test, Figure 1A). Likewise, *CYSLTR2* expression was significantly decreased in CRC tumor tissues compared with corresponding normal tissues (*p* ≤ 0.00001, paired t-test, Figure 1B). These data are significant, as high *CYSLTR1* expression was correlated with poor prognosis in CRC patients. *CYSLTR1* expression significantly separated the good and poor prognosis groups for overall survival in the TCGA-COADREAD cohort with a hazard ratio (HR) of 1.87 (95% CI = 1.04–3.37, *p* = 0.03, Figure 1C). Low expression of *CYSLTR2* was not significantly correlated with the poor prognosis group in CRC patients (Figure 1D).

On the other hand, high *CYSLTR1* expression was also significantly correlated with poor prognosis, with an HR of 1.54 (95% CI = 1.02–3.59, *p* = 0.05, Figure 1E) for disease-free survival (DFS). In accordance with the overall survival for *CYSLTR2* expression, the DFS Kaplan–Meier curve exhibited the opposite result; a high expression of *CYSLTR2* was significantly correlated with poor prognosis in the TCGA-COADREAD cohort (HR = 1.84, 95% CI = 1.15–4.82, *p* = 0.04, Figure 1F). After correlating the M-values for the CpG probes of *CYSLTR1* (cg00813999 and cd26848126) and *CYSLTR2* (cg16886259 and cg16299590) with *CYSLTR1* and *CYSLTR2* gene expression, we observed a significant reduction in negative M-values for high expression of the *CYSLTR1* gene and a significant increase in positive M-values for low expression of the *CYSLTR2* gene. Hence, the *CYSLTR1* and *CYSLTR2* genes were hypomethylated and hypermethylated in CRC tumors, respectively, and significantly controlled the associated gene expression (Figure 1G).

**Figure 2:**
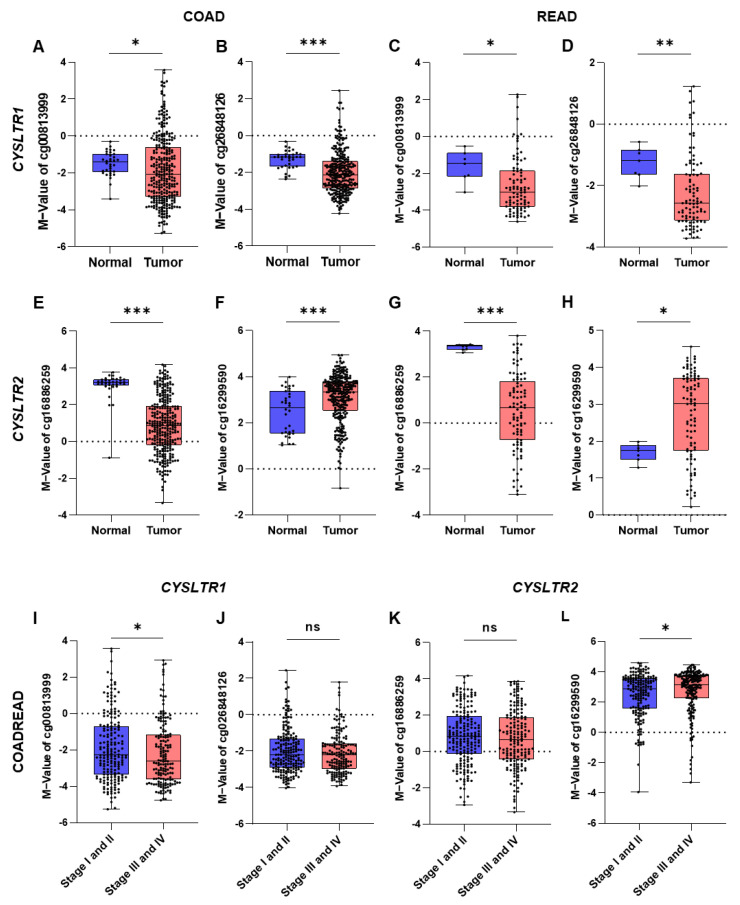
Box-and-whisker plots of the M−value distribution for CpG probes between normal and tumor samples in the TCGA−COADREAD cohort. The M−value distribution between normal and tumor samples for cg00813999 (**A**,**C**) and cg26848126 (**B**,**D**) in colon (COAD) and rectal (READ) cancer. The M−value distribution between normal and tumor samples for cg16886259 (**E**,**G**) and cg16299590 (**F**,**H**) in colon (COAD) and rectal (READ) cancer. The M−value distribution between early and advanced stages for cg00813999 (**I**), cg26848126 (**J**), cg16886259 (**K**) and cg16299590 (**L**) in colorectal (COADREAD) cancer. * *p* < 0.05; ** *p* < 0.01; *** *p* < 0.001; ns = not significant.

There was a significant decrease in the negative M values for the two CpG probes for *CYSLTR1* in colon tumors (cg00813999 and cd26848126, Figure 2A,B) and rectal tumors (cg16886259 and cg16299590, Figure 2C,D) compared with matched normal samples. However, a significant decrease in positive M-values for the CpG probe cg16886259 (Figure 2E,G) and an increase in positive M-values for the CpG probe cg16299590 (Figure 2F,H) for *CYSLTR2* were observed in the colon and rectal tumors compared with matched normal samples. On the other hand, the M-values for cg00813999 and cd16299590 probes were significantly decreased and increased in the late TNM stages (stage III and IV) compared with early TNM stages (stage I and II), respectively (Figure 2I,L), but the other two probes (cg026848126 and cg16886259) did not achieve a significant level (Figure 2J,K).

**Figure 3:**
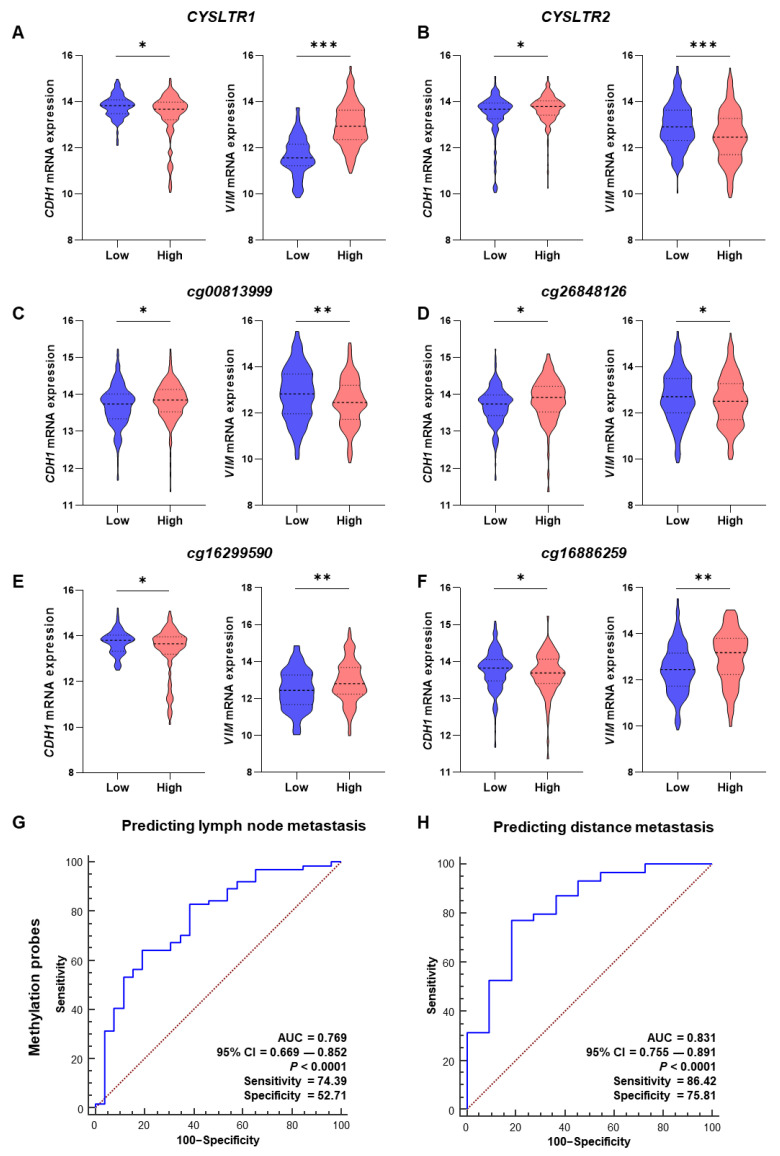
Correlation of EMT marker (*CDH1* and *VIM*) expression and *CYSLTR1* and *CYSLTR2* gene expression and their CpG probes in the TCGA-COADREAD cohort. (**A**) Correlation between *CDH1* and *VIM* gene expression with high and low *CYSLTR1* gene expression. (**B**) Correlation between *CDH1* and *VIM* gene expression with high and low *CYSLTR2* gene expression. (**C**–**F**) Correlation between *CDH1* and *VIM* gene expression with high and low M values for the CpG probes of *CYSLTR1* (cg00813999 and cg26848126) and *CYSLTR2* (cg16299590 and cg16886259) genes. ROC–AUC curve for the prediction of lymph node metastasis (**G**) and distant metastasis (**H**) using the M values for CpG probes of *CYSLTR1* and *CYSLTR2* genes. * *p* < 0.05; ** *p* < 0.01; *** *p* < 0.001.

There was a significant correlation between the mRNA expression of the EMT (epithelial–mesenchymal transition) markers *CDH1* (E-cadherin) and *VIM* (vimentin) and high *CYSLTR1* gene expression in tumor samples. *CDH1* and *VIM* gene expression were significantly reduced and increased, respectively, in tumor samples with high *CYSLTR1* gene expression (Figure 3A). However, the opposite was true for *CYSLTR2* gene expression (Figure 3B). *CDH1* and *VIM* gene expression were significantly decreased and increased in the low M-value groups for both CpG probes of *CYSLTR1* (cg00813999 and cd26848126, Figure 3C,D). Interestingly, the opposite result was obtained for both CpG probes of *CYSLTR2* (cg16886259 and cg16299590, Figure 3E,F). Hence, these results indicate a positive association between CpG probe methylation and *CYSLTR1* and *CYSLTR2* gene expression to control EMT markers in CRC. Next, we checked the prediction ability of lymph node metastasis (LNM, Stages II and III) and distant metastasis (ME Stage IV), we performed ROC–AUC (receiver operating curve–area under the curve) analysis using the multivariate logistic regression probability scores of the four probes. Surprisingly, we achieved a significantly high AUC value for both models (for LNM, AUC = 0.769, Figure 3G; and for ME, AUC = 0.831, Figure 3H) with high sensitivity.

**Figure 4:**
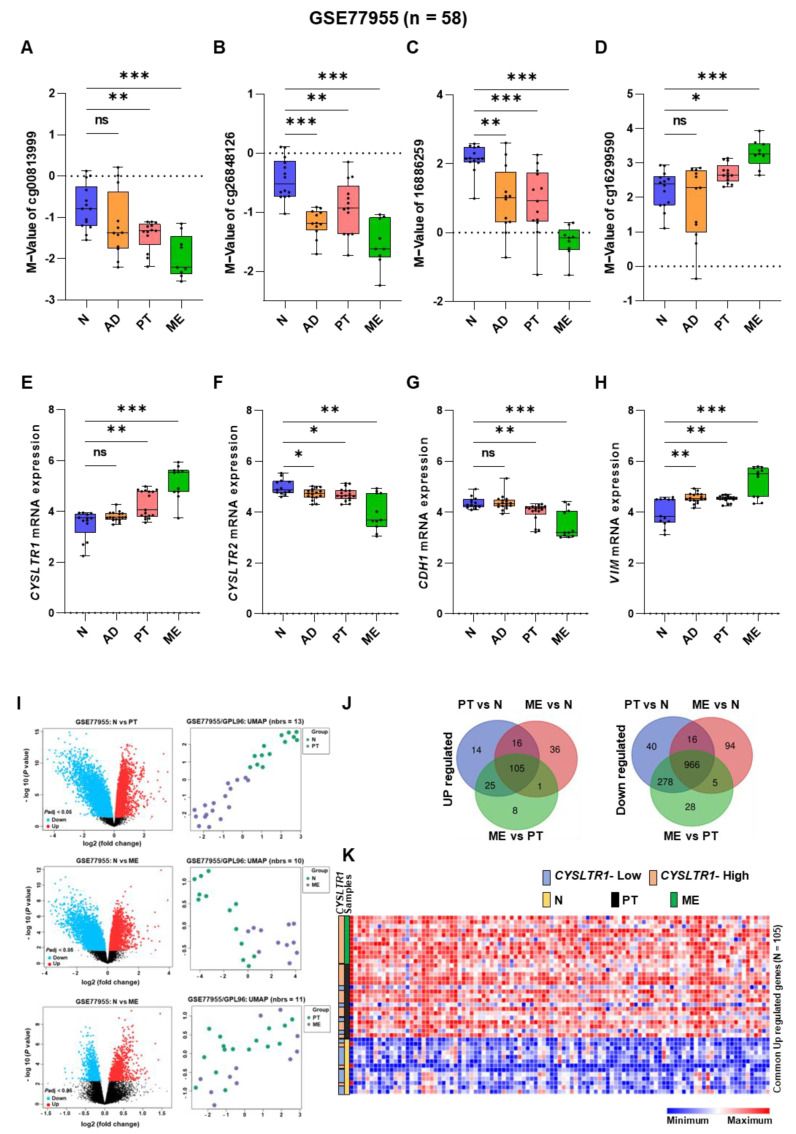
M-value distribution for the CpG probes of the *CYSLTR1* and *CYSLTR2* genes and their expression in matched normal (N), adenoma (AD), primary tumor (PT) and distant metastasis (ME) samples in the GSE77955 CRC cohort. (**A**–**D**) M-value distribution for the CpG probes in the N, AD, PT and ME samples. Relative expression of *CYSLTR1* (**E**) and *CYSLTR2* (**F**) genes between the N, AD, PT and ME samples. Relative expression of *CDH1* (**G**) and *VIM* (**H**) genes between the N, AD, PT and ME samples. (**I**) Volcano plots and UMAP plots for the differentially expressed genes (DEGs) between PT vs. N, ME vs. N and ME vs. PT samples. (**J**) Venn diagram for the up-and downregulated genes from the DEGs between PT vs. N, ME vs. N and ME vs. PT samples. (**K**) Heatmap for the commonly upregulated gene expression in N, PT and ME samples. * *p* < 0.05; ** *p* < 0.01; *** *p* < 0.001.

To validate our results from the TCGA-COADREAD datasets, we used the GEO database (GSE77955 dataset) with adenoma, matched normal, primary tumor and distant metastasis tissues. Interestingly, the negative M-value for the CpG probe for *CYSLTR1* genes was significantly lower for primary tumors than for adenoma and matched normal tissues. Moreover, it was further decreased in the metastatic specimens compared with the primary tumor (Figure 4A,B). The result was similar for the CpG probe cg16886259 (Figure 4C) for the *CYSLTR2* gene, but the other probe, cg16299590 (Figure 4D), exhibited the opposite result to that observed in the TCGA-COADREAD cohort.

*CYSLTR1* and *CYSLTR2* expression were significantly higher and lower in the primary tumor samples than in the adenoma and normal samples, but it was higher and lower in the metastatic specimens than in the primary tumor samples, respectively (Figure 4E,F). Whereas *CDH1* expression was gradually decreased in primary tumor and metastasis specimens compared with adenoma and normal samples, the opposite was true for *VIM* expression (Figure 4G,H). Hence, *CYSLTR1* and *CYSLTR2* expression influenced tumor metastasis through the alteration of EMT marker expression (*CDH1* and *VIM*). *CYSLTR1*/2 gene expression was controlled by the methylation of CpG probes, specifically cg00813999 and cg26848126 for *CYSLTR1* and cg16299590 for *CYSLTR2*.

We found 13 983, 12 925 and 5 169 genes from the differential gene expression (DGE) analysis between primary tumor vs. normal (PT vs. N), distant metastasis vs. normal (ME vs. N) and distant metastasis vs. primary tumor (ME vs. PT) samples, respectively (cutoff: adjusted *p* ≤ 0.05 and log fold change more than ± 1) (Figure 4I). The UMAP plots for normal vs. tumor and normal vs. metastasis exhibited two distinct separate clusters for the group of samples used for DGE, but the tumor vs. metastasis group did not separate the samples distinctly (Figure 4I). We found 105 upregulated and 966 downregulated genes that are common between PT vs. N, ME vs. N and ME vs. PT by the DGE analysis (Figure 4J). *CYSLTR1* gene expression was significantly high in tumor and metastasis samples, and the 105 common upregulated genes were also significantly high in these groups; hence, the expression of these genes was positively correlated with *CYSLTR1* expression (Figure 4K, Supplementary Table S1).

**Figure 5:**
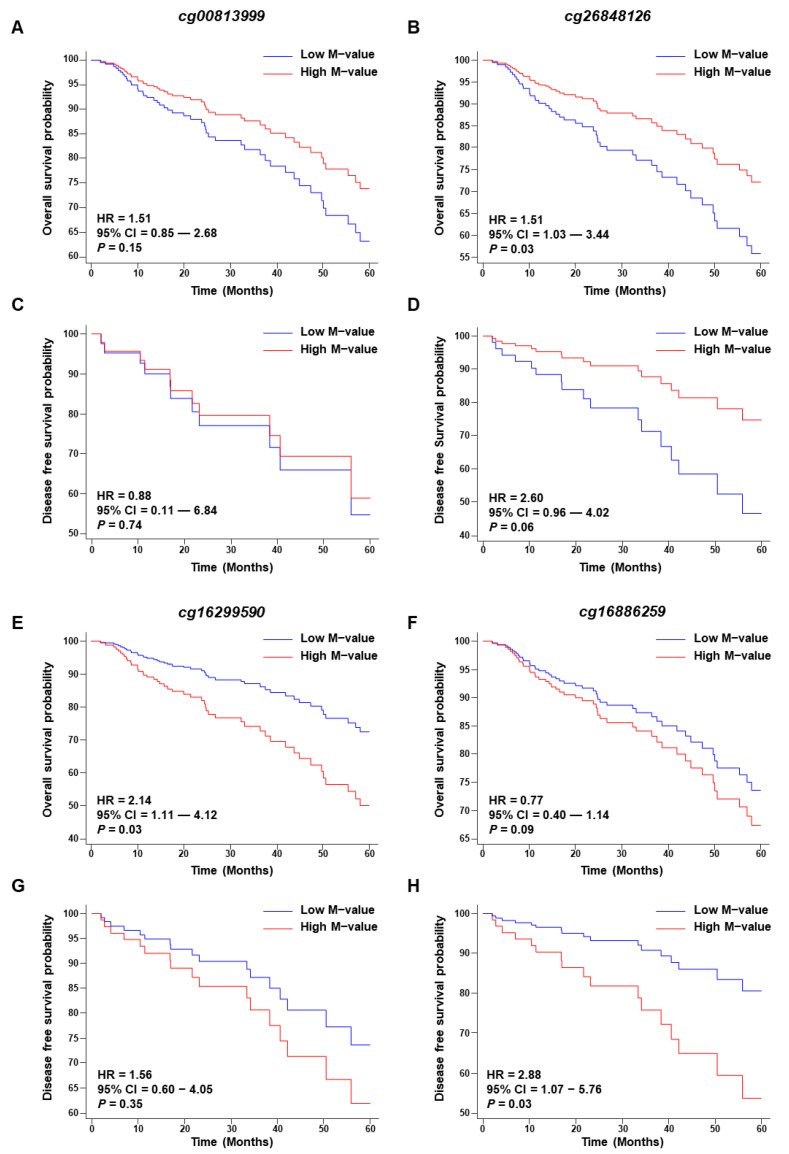
Kaplan–Meier plot for the CpG probes. Five-year overall and disease-free survival plots for CpG probes cg00813999 (**A**,**C**) and cg26848126 (**B**,**D**) for the *CYSLTR1* gene. Five-year overall and disease-free survival plots for CpG probes cg16299590 (**E**,**G**) and cg16886259 (**F**,**H**) for the *CYSLTR2* gene.

We achieved a significant hazard ratio (HR) in the overall survival analysis for the CpG probe cg26848126 (HR = 1.51, 95% CI = 1.03–3.44, *p* = 0.03), whereas the other CpG probe (cg00813999) for *CYSLTR1* did not achieve a significant *p* value (Figure 5A,B). The low M-value significantly separated the poor prognosis group for five years of OS prediction. On the other hand, the HR was not significant for five years of DFS for either CpG probe, although the low M-value of cg26848126 could separate the poor prognosis group with a *p* = 0.06 (Figure 5C,D). Interestingly, we observed the opposite trend for the CpG probe of *CYSLTR2*. A high M-value for both probes (cg16299590 and cg16886259) was positively correlated with poor prognosis in five years of overall survival; although cg16299590 achieved a significant HR (HR = 2.14, 95% CI = 1.11–4.12, *p* = 0.03, Figure 5E), the other probe did not achieve a significant *p* value (HR = 0.77, 95% CI = 0.44–1.14, *p* = 0.09, Figure 5F). In the case of the five-year DFS prediction, a high M-value of the CpG probes of the *CYSLTR2* gene was positively correlated with poor prognosis (Figure 5G,H), although cg16886259 was only significant for DFS assessment (HR = 2.88, 95% CI = 1.07–5.76, *p* = 0.03, Figure 5H).

**Figure 6:**
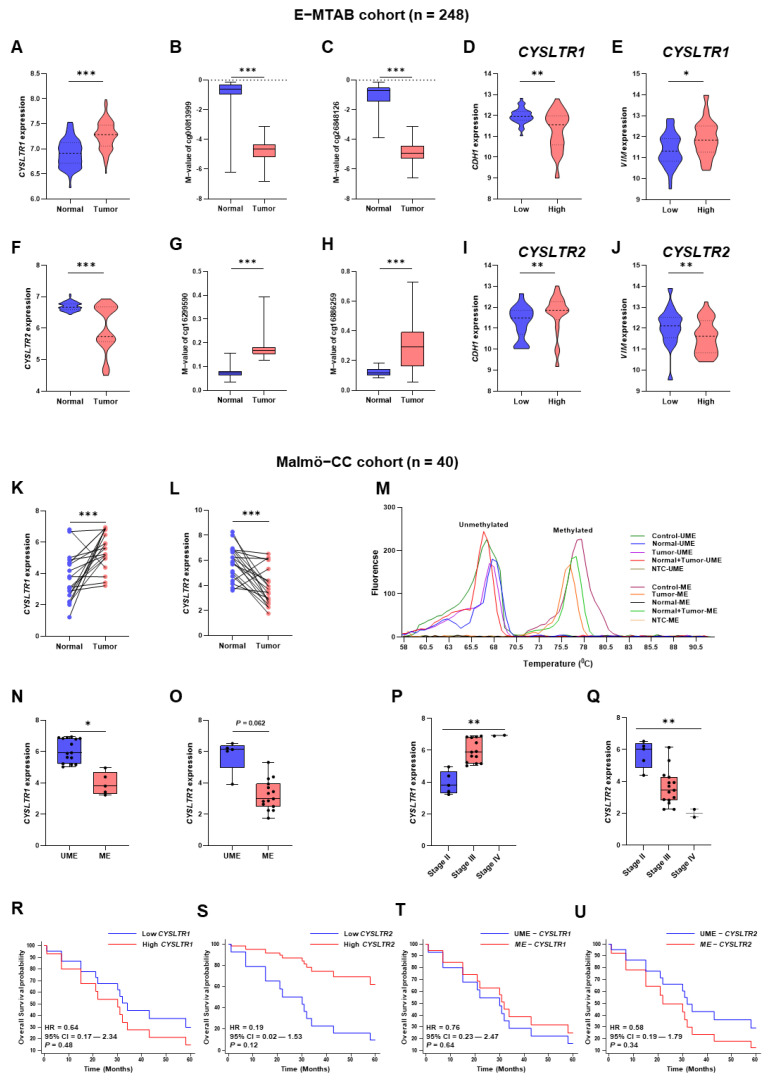
Expression and DNA methylation of *CYSLTR1* and *CYSLTR2* genes in the E-MTAB in silico and Malmö-CC clinical cohort. Relative expression of *CYSLTR1* (**A**) and M-value distribution of cg00813999 (**B**) and cg26848126 (**C**) between normal and tumor samples, *CDH1* (**D**), *VIM* (**E**) gene expressions in low- and high-expressed *CYSLTR1* groups of patients in E-MTAB cohort. Relative expression of *CYSLTR2* (**F**) and M-value distribution of cg16299590 (**G**) and cg16886259 (**H**) between normal and tumor samples, *CDH1* (**I**), *VIM* (**J**) gene expressions in low- and high-expressed *CYSLTR2* groups of patients in E-MTAB cohort. Relative expression of *CYSLTR1* (**K**) and *CYSLTR2* (**L**) gene expression between normal (N) and primary tumor (PT) samples in the Malmö cohort. (**M**) Quantitative methylation-specific PCR melting curve analysis in N and PT samples from the Malmö cohort. *CYSLTR1* (**N**) and *CYSLTR2* (**O**) gene expressions in unmethylated and methylated (*CYSLTR1* and *CYSLTR2*) patient groups in the Malmö cohort. *CYSLTR1* (**P**) and *CYSLTR2* (**Q**) gene expression in different stage (stage II, III and IV) tumor samples in the Malmö cohort. Five year overall survival plots for *CYSLTR1* (**R**), *CYSLTR2* (**S**), *CYSLTR1*—unmethylated and methylated (**T**) and *CYSLTR2*—unmethylated and methylated (**U**) patient samples in the Malmö cohort. HR—hazard ratio, CI—confidence interval, UME—Unmethylated, ME—Methylated, * *p* < 0.05; ** *p* < 0.01; *** *p* < 0.001.

The *CYSLTR1* and *CYSLTR2* gene expressions and methylation were validated in an additional in silico cohort (E-MTAB), with the transcriptome and genome-wide methylation sequencing for primary tumor and normal samples (Figure 6A–J). In tumor samples, *CYSLTR1* (Figure 6A) was significantly upregulated, but the opposite was true for *CYSLTR2* (Figure 6F) expression. The M values of cg00813999 (Figure 6B) and cg26848126 (Figure 6C) CpG probes were significantly lower in the tumor samples than normal. Conversely, *CDH1* (Figure 6D) and *VIM* (Figure 6E) expression were low and high in patients with high *CYSLTR1* gene expression, respectively. The M values of cg16299590 (Figure 6G) and cg16886259 (Figure 6H) CpG probes for the *CYSLTR2* gene were significantly high in tumor samples than in normal areas. Interestingly, *CYSLTR2* gene expression was low in patients with low *CDH1* (Figure 6I) and high *VIM* (Figure 6J) expression.

**Figure 7:**
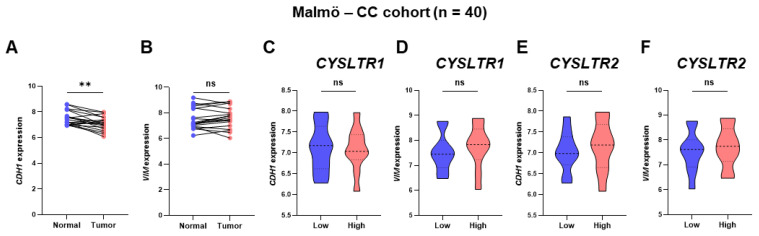
Expression of the EMT markers *CDH1* and *VIM* in patient samples with low and high expression of *CYSLTR1* and *CYSLTR2* genes in the Malmö–CC clinical cohort. Relative expression of *CDH1* (**A**) and *VIM* (**B**) genes between normal and primary tumor samples. *CDH1* (**C**) and *VIM* (**D**) gene expressions in low and high *CYSLTR1* groups of patients. *CDH1* (**E**) and *VIM* (**F**) gene expressions in low and high *CYSLTR2* group of patients. ** *p* < 0.01; ns = non-significant.

The results were validated in a CC patient cohort (Malmö-CC) [14], consisting of twenty paraffin-embedded normal tissues and twenty matched primary tumor tissues. The *CYSLTR1* gene was significantly upregulated in tumors and the *CYSLTR2* gene was significantly downregulated in tumors compared with normal samples (Figure 6K,L). The promotor region methylation status of the *CYSLTR1* and *CYSLTR2* genes were validated using quantitative methylation-specific melting curve analysis and agarose gel electrophoresis using a specific set of primers (Figure 6M, Supplementary Figure S2A,B). *CYSLTR1* and *CYSLTR2* gene expressions were higher in unmethylated samples than in methylated samples in the Malmö-CC cohort (Figure 6N,O). Interestingly, the CC samples from the high-risk group based on the *CYSLTR1* gene expression were unmethylated and highly expressed; however, the CC samples in the high-risk group based on *CYSLTR2* gene expression were methylated and less expressed. The *CYSLTR1* and *CYSLTR2* gene expressions gradually increased and decreased in stage II, III and IV primary tumor samples, respectively (Figure 6P,Q). The Malmö-CC cohort demonstrated high expression of *CYSLTR1* (Figure 6R) and low expression of *CYSLTR2* (Figure 6S), while unmethylated *CYSLTR1* (Figure 6T) and methylated *CYSLTR2* (Figure 6U) genes were observed in the poor prognosis group, although at a statistically non-significant level. *CDH1* gene expression was significantly downregulated in the tumor samples compared to normal areas, while *VIM* expression did not differ between tumors and normal areas (Figure 7A,B). Interestingly, *CDH1* expression was lower and *VIM* expression was higher in the tumor samples with high *CYSLTR1* expression, although the statistical significance was not achieved, possibly due to the smaller number of patients (Figure 7C,D). On the other hand, *CDH1* and *VIM* expression were higher in tumor samples with high expression of the *CYSLTR2* gene (Figure 7E,F). We next used CC SW620 cell-derived colonospheres with or without CysLT_1_R expression. The colonospheres showed a decrease in E-cadherin expression after LTD_4_ stimulation, which was not observed in the CysLT_1_R, knockdown cells. No significant changes were observed of the mesenchymal marker vimentin (Supplementary Figure S3A–C).

## 3. Discussion

Our goal was to determine the influence of the methylation profile of the CysLTRs receptors on their gene expression and their association with prognosis and metastasis development in CRC patients. We successfully identified methylated CpG probes for CysLTRs that could influence gene expression in CRC. Furthermore, the gene expression and methylation profiles for the CysLTRs are one of the strongest prognostic indicators and metastasis predictors for CRC patients in the TCGA-COADREAD cohort. To further highlight the clinical significance of our findings, we validated the results using the well-structured GSE77955 cohort with normal tissue, primary tumor and distant metastasis samples. The results were further validated using an additional in silico cohort (EMBL—EBI, E-MTAB-8148), which included normal tissues and primary tumors and FFPE tissue-based CC clinical cohort (Malmö-CC), which includes both normal tissues and primary tumors.

The role of CysLTRs (CysLT_1_R and CysLT_2_R) has been well reported for the development and metastasis of different types of cancer [34]. We previously showed that high CysLT_1_R expression in CRC patients was associated with poor prognosis and was positively correlated with nuclear β-catenin and negatively correlated with membrane β-catenin, which is associated with poor prognosis for CRC patients [14]. Although previous studies have demonstrated the involvement of CysLTRs in various cancers, their precise role in cancer pathogenesis and the molecular mechanisms underlying their methylation profile remain unclear. We identified that CRC tumors exhibited higher *CYSLTR1* gene expression than matched normal tissues, whereas the opposite was true for *CYSLTR2* expression. This is supported by the result from Magnusson et al. that the expression of CysLT_1_R was higher in colon tumor tissues than in matched normal mucosa [14]. TCGA-COADREAD data also suggests that high expression of the *CYSLTR1* gene and low expression of the *CYSLTR2* gene are associated with a poor prognosis in CRC patients. This finding was supported by the data generated from our earlier publications at the protein level using the patient CRC tumor microarray (TMA), which showed that high protein expression of CysLT_1_R was associated with poor prognosis and that low protein expression of CysLT_2_R was positively correlated with poor prognosis in CRC patients [14]. Notably, high expression of CysLT_1_R was associated with poor prognosis in CRC patients and reduced survival and stemness in colorectal and breast cancer [14,16,17], while CysLT_2_R has been reported to have an antitumorigenic effect in CRC patients and cell lines [14,35].

Our study is the first to show that the methylation and gene expression profiles for *CYSLTR1*/*CYSLTR2* receptors together to investigate their role in colorectal cancer progression and metastasis using three independent in silico datasets and one clinical cohort. DNA CpG methylation is usually associated with a closed state of chromatin and has been well-accepted as an important mechanism for maintaining gene expression and pathway alteration in diseases [36,37]. Usually, DNA methylation and gene expression are negatively correlated with each other, but very few genes have been reported, and the correlation direction is both positive and negative [38]. It is important to determine the influence of methylation profiles on cancer-associated gene expression. To prove this hypothesis for CysLTRs, we used three independent CRC datasets (TCGA-COADREAD, GSE77955 and E-MTAB-8148), which included methylation and gene expression profiles for each patient.

Here, we investigated the interplay between CpG methylation and gene expression for CysLTRs in CRC progression, metastasis, and patient prognosis. We used the GSE77955 dataset genome-wide deep sequencing to compare the methylomes and transcriptomes of primary CRCs and CRC liver metastases. The methylation profile for *CYSLTR1* genes was used only to establish lung function in asthmatic individuals exposed to traffic-related air pollution and not for any cancer [39]. Although the role of CysLTRs in relation to the development and metastasis of different cancers has been well established, it is important to determine the effect of the methylation profile of CpG probes for CysLTRs on gene expression, cancer progression and metastasis. Interestingly, the high expression of the *CYSLTR1* gene was positively correlated with the more hypomethylated patient group, and the low expression of the *CYSLTR2* gene was significantly correlated with the more hypomethylated patient group.

Based on the annotations from UCSC, the CpG probes for CRC were located on the CpG island (promoter region) and the shore of the *CYSLTR1* and *CYSLTR2* genes, respectively (Supplementary Figure S4). Among all the clinicopathological factors, a history of colon polyps was significantly correlated with *CYSLTR1* gene expression in CRC patients, but the sample type (metastasis, normal and primary tumor) was significantly correlated with *CYSLTR1* and *CYSLTR2* gene expression in colon and rectal cancer patients (Supplementary Figure S4). We observed a significant number of mutations in the *CYSLTR1* and *CYSLTR2* genes in CC, whereas the *CYSLTR1* gene was not mutated in rectal cancer patients. CysLTRs expression was negatively correlated with copy number variation in CRC patients.

The M-values for the CpG probes of *CYSLTR1* (cg00813999 and cg16299590) were significantly decreased and increased, respectively, for advanced-stage patients compared with early-stage patients. Hence, these CpG probes were significantly associated with CRC progression. The activation of LTD_4_–CysLT_1_R signaling is well-reported to promote cell proliferation and survival through multiple pathways [7,40]. Furthermore, our previous findings showed increased expression of CysLT_1_R in patients with CC and the inhibition of LTD_4_ signaling by blocking CysLT_1_R receptor-induced apoptosis in CC cells [12,41,42,43]. However, the methylation profile of CysLTRs has not been studied for cancer progression and metastasis. Hence, it is necessary to fill this gap to establish the role of CysLTRs in cancers. Among the most widely used drugs that block the actions of CysLTRs are also those commonly used to treat allergic asthma [44,45]. In addition to its role in asthma, the leukotriene pathway is known to contribute to cancers and tumor-mediated immune suppression [46]. Furthermore, a comprehensive study from Taiwan with two million subjects reported that the use of a CysLT_1_R antagonist in asthma patients is associated with a significantly decreased risk of cancer in a dose-dependent manner [9]. A recent study from the United States with more than five million asthma patients (with or without CysLT_1_R antagonist treatment) concluded that antagonists reduced the risk of lung cancer by 22% [47].

The disturbance of the E-cadherin–catenin adhesion complex is one of the main events in the early and late stages of cancer [48]. The inhibition of GSK-3β leads to the downregulation of E-cadherin, which can also lead to the cytoplasmic mobilization of β-catenin [49,50]. Relatively little is known about the ability of leukotrienes to regulate tumor cell migration and invasion, but LTB_4_ was shown to inhibit metastatic spread to the liver and other organs in an in vivo study of pancreatic cancer [51], and previous results from our laboratory suggested that LTD_4_ could induce the cell invasion via modulating the expression of EMT markers [22]. As a result of LTD_4_ treatment, E-cadherin (*CDH1*) was downregulated in the plasma membrane, and cell–cell contacts were reduced, whereas montelukast restored the E-cadherin expression to the control levels [22]. Lukic et al., demonstrated that exosomes prepared from lung cancer patient pleura exudates promoted the migration of both A549 lung cancer cells and primary lung cancer cells via CysLTs, whereas the CysLT_1_R antagonist montelukast blocked this migration [52]. Interestingly, we observed a significant reduction in E-cadherin in CRC patients with high expression of *CYSLTR1* and low expression of *CYSLTR2*, while *VIM* expression showed the opposite trend. Moreover, *CDH1* and *VIM* expression was significantly increased and decreased, respectively, in the methylated *CYSLTR1* gene. However, it was oppositely regulated for the methylated *CYSLTR2* gene. Hence, EMT might be regulated through the methylation of CysLTRs, ultimately controlling their expression. E-cadherin expression was significantly increased after inhibiting the LTD_4_ signaling pathway and β-catenin expression in a SW480 CC cell line, followed by a reduction in cancer cell migration [53]. In this study, we successfully estimated the prediction ability for lymph node metastasis (Figure 3G) and distant metastasis (Figure 3F) in a group of patients using the methylation of the CysLTRs. Therefore, CpG probe methylation of CysLTRs could be a valuable marker for detecting a group of CRC patients with lymph node and distant metastasis. Moreover, in another report, the direct association of CysLT_1_R with CRC metastasis was established in a zebrafish model, with less metastatic foci found in the montelukast-treated group compared to the only-LTD_4_-treated group [54].

We found a similar trend for methylation and expression of CysLTRs for the metastasis group of patients in the GSE77955 patient cohort. Thus, the distant metastasis samples exhibited reduced methylation and high expression of CysLT_1_R and high methylation and reduced expression of CysLT_2_R. This finding provides direct evidence of the relationship between CysLTRs and metastasis in CRC patients. Moreover, E-cadherin was significantly lower, and vimentin was higher in metastasis samples than in primary tumors for this cohort. Interestingly, the differentially upregulated common genes for the T vs. N, M vs. N and M vs. N groups exhibited higher expression of *CYSLTR1* in primary tumors than in matched normal samples, whereas it was further increased for distant metastasis samples. Therefore, *CYSLTR1* expression might control the expression of other genes involved in the development of metastasis in CRC patients. The methylation profile for CpG probes for CysLTRs also significantly predicted the OS and DFS of CRC patients. The OS curves for cg26848126 (*CYSLTR1*) and cg16299590 (*CYSLTR2*) were significant, and the DFS curves for cg16886259 (*CYSLTR2*) were significant for CRC patients in the TCGA-COADREAD cohort. Hence, the methylation of the *CYSLTR1* and *CYSLTR2* genes could influence OS and DFS in CRC patients, respectively.

Due to the small number of samples in the Malmö–CC clinical cohort, there was no significant correlation between *CDH1* and *VIM* expression and *CYSLTR1* and *CYSLTR2* expression. However, the LTD_4_-treated SW620 CC cell-derived colonospheres model exhibited less expression of E-cadherin (*p* ≤ 0.01), and *CYSLTR1* knockdown did not significantly increase the E-cadherin expression (Supplementary Figure S3A,B), whereas the expression of vimentin was not significantly changed after LTD_4_ treatment or *CYSLTR1* knockdown. As we reported in our previous publications, E-cadherin was decreased by LTD_4_ in HCT-116 CC cells [21], and one of the EMT markers, MMP-9, was also induced by LTD_4_ in SW480 CC cells [22]. Considering the complexity of the epithelial-to-mesenchymal transition state in cancer, our observations provide some insights into the involvement of methylation and gene expression of CysLT_1_R in preparing cells for the transition state without controlling the whole phenomenon.

## 4. Materials and Methods

### 4.1. Patient Cohorts

This study included four CRC patient cohorts with a total of 762 patients. These cohorts included patients from three public datasets—the in silico discovery cohort from the Cancer Genome Atlas [TCGA-COADREAD; primary tumor (PT) = 375 and matched normal (N) = 41], the two in silico validation cohorts from the Gene Expression Omnibus (GEO; GSE77955; N = 13; PT = 17; matched distant metastasis, ME = 11 and adenoma from separate patients, AD = 17) and the European Molecular Biology Laboratory—European Bioinformatics Institute (EMBL—EBI, E-MTAB-8148, N = 32 and PT = 216) and one patient-based clinical validation cohort from the Malmö—colon cancer (Malmö-CC; N = 20; PT = 20). All the in silico cohorts are unique because of the availability of genome-wide methylation and transcriptome profiles for all the patients in these cohorts (Table 1).

### 4.2. Analysis of DNA Methylation in the Cancer Genome TCGA and GSE Cohort

DNA methylation and clinical data for colorectal cancer (COADREAD) were collected from TCGA (International Cancer Genome Consortium) [55]. The data were downloaded from UCSC Xena (http://xena.ucsc.edu, accessed on 23 April 2022) [56]. The DNA methylation profile was measured experimentally using the Illumina Infinium HumanMethylation 450k platform (Illumina, San Diego, CA, USA), which contains 485 577 CpG sites. The methylation level was expressed as β and M-values. Poorly performing probes, cross-reactive probes, and SNP probes were excluded from our data processing. The R function “BMIQ type-II probe normalization” was used to normalize the data between arrays. For validation, the methylation profiles of 58 matched normal, primary tumor and distant metastasis samples were collected from the GSE77955 datasets [57]. The β values of methylation sites with more than 10% missing values were deleted. The remaining missing values were estimated by the k-nearest neighbor (KNN) estimation method. The “limma” package [58] was used to calculate the methylation difference. The sites with an FDR < 0.05 and an absolute β value difference > 0.2 were considered to be differentially methylated. For the correlation analysis of DNA methylation and gene expression, we used the R package “ChAMP” to map the sites assigned to a gene. The Pearson correlation test was used (a correlation coefficient > 0.3 and a *p* < 0.05 were considered to be significant). The correlation coefficients of DMSs were obtained by the Pearson correlation test, and the R package “corplot” was used to plot the correlation between DMSs. The average β and M-values in the promoter and body regions of each gene were calculated (Figure 6A). Positive M values = more molecules methylated than unmethylated, while negative M values are the opposite.

### 4.3. Analysis of Gene Expression in the Cancer Genome TCGA and GSE Cohorts

**Figure 8:**
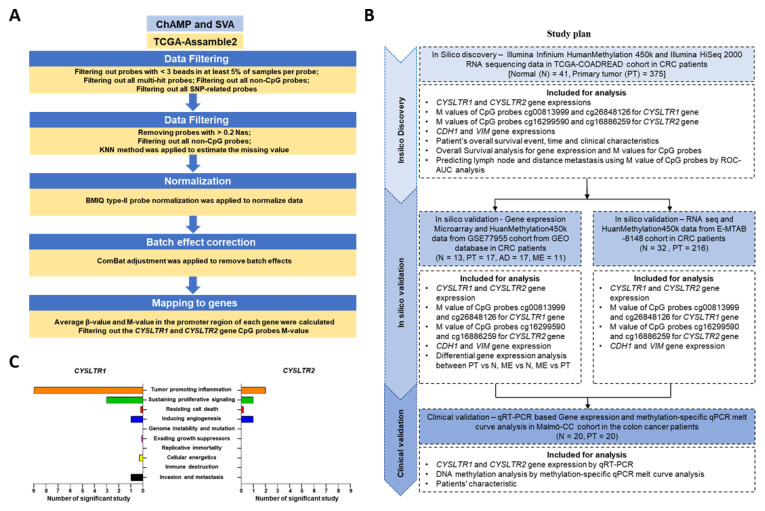
(**A**) Flowchart for CRC-specific DNA methylation data collection from TCGA-COADREAD dataset. (**B**) Flowchart for study designing, normal—N, adenoma—AD, primary tumor—PT and distant metastasis—ME. (**C**) The role of *CYSLTR1* and *CYSLTR2* in different cancer hallmarks based on different datasets and publications (based on the Cancer Hallmarks Analytics Tool (CHAT); http://chat.lionproject.net, accessed on 8 February 2022).

Gene expression and clinical data for colorectal cancer (COADREAD) were collected from TCGA. The data were downloaded from UCSC Xena (http://xena.ucsc.edu, accessed on 23 April 2022) [56]. The gene expression profile was measured experimentally using the Illumina HiSeq 2000 RNA sequencing platform (Illumina, San Diego, CA, USA). The mRNA expression levels, measured by reads per million mRNA mapped (RPM), were first log2 transformed. We checked the expression of genes that reached significance (*p* ≤ 0.05) and log2 fold change >±1. The differentially regulated genes were represented as upregulated and downregulated in the volcano plot for the GSE77955 dataset. The validation cohort was used to identify differentially regulated cancer and metastasis-related genes in the three groups, matched normal (N) vs. primary tumor samples (PT), PT vs. distant metastasis (ME) and N vs. ME, in CRC patients after performing the “limma”-based differential gene expression (DGE) analysis. Finally, the associated CpG probes and the gene expression profile for the *CYSLTR1* and *CYSLTR2* genes were filtered and used for further analysis (Figure 8A). A detailed flowchart for study designing, included with the analysis and sample information for each cohort are explained in Figure 8B. Two cancer-related receptor genes for CysLT were selected based on gene ontology and cancer hallmark databases (Figure 8C).

### 4.4. MSP Primers Designing for DNA Methylation Analysis

CpG sites were studied via the synthesis of oligonucleotide fragments (primers) representing the bisulfite-modified *CYSLTR1* and *CYSLTR2* gene sequences from Integrated DNA Technologies (Supplementary Table S2). Specifically designed primers using the MethPrimer tool (Li Lab, Dongcheng, Beijing, China) [59] for melt curve analysis amplified methylated as well as unmethylated bisulfite-modified DNA, but not unmodified DNA. To increase the likelihood of amplification of only bisulfite modified template, the primer contained at least one T corresponding to a non-CpG C at the 3’-end of the forward primers. As far as possible, CpGs were avoided, but, when necessary, should be placed at the 5’-end of the primer with a degenerate base. These allow both methylated and unmethylated template amplification. Primers had limited self-complementarity between pairs which was analyzed using OligoAnalyzer™ Tool (https://eu.idtdna.com/pages/tools/oligoanalyzer, accessed on 14 August 2022).

### 4.5. DNA/RNA Extraction from FFPE Tissue and Bisulfite Modification of Extracted DNA

Nucleic acids (DNA and RNA) were extracted from formalin-fixed paraffin-embedded (FFPE) matched normal and tumor specimens using the previously published protocol after some modifications [60]. Extracted genomic DNA (1 µg) was bisulfite modified using the Epitect Fast Bisulfite Conversion kit (Qiagen, Hilden, Germany). Extracted RNA (1 µg) was converted to cDNA using the RevertAid H Minus First Strand cDNA Synthesis Kit (Thermo Fisher Scientific Inc., Rochester, NY, USA).

### 4.6. DNA Methylation by qPCR and Melt Curve Analysis

Melt curve analysis was used to identify methylated *CYSLTR1* and *CYSLTR2* genes using the previously published protocol after some modifications [61]. Bisulfite-modified DNA (2 μL) was amplified using Maxima SYBR Green/ROC qPCR master Kit (Thermo Fisher Scientific, Inc. Rochester, NY, USA) containing a final concentration of 0.5 μM of each primer in a final reaction volume of 15 μL. Both primers and PCR conditions were specific for bisulfite-modified DNA and did not produce amplification of unmodified DNA. Every run included fully methylated, fully unmethylated, and no template control. The PCR was performed using a Stratagene Mx3005P qPCR (Agilent Technologies, Santa Clara, CA, USA) with a 95 °C activation step for 10 min; 95 °C for 30 s, 55 °C for 60 s for 40 cycles; and a final extension step of 72 °C for 5 min. In order to melt the PCR product, we increased the temperature from 58 to 92 °C in increments of 0.5 °C, waited for 30 s at the first step and for 5 s at each subsequent step, and acquired fluorescence for each temperature increment.

### 4.7. qPCR for CYSLTR1, CYSLTR2, CDH1 and VIM Gene Expression

qPCR was used to evaluate the expression profiles of *CYSLTR1* and *CYSLTR2* genes using the Maxima Probe/ROX qPCR Master Mix (Thermo Fisher Scientific Inc., Rochester, NY, USA) and Maxima SYBR Green/ROC qPCR master Kit (Thermo Fisher Scientific Inc., Rochester, NY, USA). TaqMan probes (Thermo Fisher Scientific Inc., Rochester, NY, USA) for the following genes were used in this study: *CYSLTR1* (Hs00929113_m1), *CYSLTR2* (Hs00252658_s1), and *HPRT1* (Hs99999909_m1) and primers for SYBR Green-based qPCR of *CDH1*, *VIM* and *GAPDH* genes are listed in Supplementary Table S2. Normalization was performed using the endogenous housekeeping gene *HPRT1* for TaqMan probes and *GAPDH* for SYBR Green. MxPro software (Agilent Technologies, Santa Clara, CA, USA) was used to quantify fold changes using the 2^−ΔΔCt^ method.

### 4.8. CRISPR-Cas9 Based Knockdown of CysLT_1_R

CRISPR-Cas9 based knockdown of *CYSLTR1* in SW620 CC cells was achieved using the protocol from Satapathy et al. [54]. Briefly, after transfection of cells with either *Cas9-CTRL* or *CRISPR-CYSLTR1* using lipofectamine, 2000, cells were subjected to antibiotic selection. *Cas9-CTRL* (sc-418922; Control *CRISPR/Cas9* Plasmid); *CRISPR-Cas9* for *CYSLTR1* (sc-416516; Santacruz Biotechnology, Heidelberg, Germany) were used for the *CYSLTR1* knockdown. Selected colonies were expanded and used for the colonosphere formation.

### 4.9. SW620 Cells Colonosphere Formation and Western Blot Analysis

SW620 CC cell-derived colonospheres were formed using the protocol described earlier [53,61]. Briefly, cells were counted after trypsinization and approximately 1000 cells were seeded per well in ultra-low attachment round bottom plates (7007; Corning Inc., Corning, NY, USA). For the formation of colonospheres, DMEM-F12 medium supplemented with L-glutamine and antibiotics was used. After 3 weeks colonospheres were collected from each well and protein was extracted using RIPA lysis buffer. Extracted protein was used for western blot analysis of the following proteins: E-Cadherin (#3195, Cell Signaling Technology, Danvers, MA, USA); vimentin (#5741, Cell Signaling Technology, Danvers, MA, USA); CysLT_1_R (NBP2-92396; Novus Biologicals, Centennial, CO, USA). α-Tubulin (sc-8035; Santa Cruz Biotechnology, Heidelberg, Germany) antibodies were used for western blot experiment [54,62].

### 4.10. Statistical Analysis and Data Visualization

Statistical analyses were performed using IBM SPSS version 20 (IBM, Chicago, IL, USA), MedCalc version 18 (MedCalc Software Ltd., Ostend, Belgium), GraphPad Prism version 8.0 (La Jolla, CA, USA) and R 3.2.4 (The R Foundation, Indianapolis, IN, United States). Statistical differences between mRNAs and various clinicopathologic factors were determined by the χ2 test. The Benjamini–Hochberg method was used to correct for multiple hypothesis testing wherever applicable. All statistical tests were two-sided, and a *p* ≤ 0.05 was considered significant. OS was defined from the day of surgery to death or the end of follow-up and was analyzed by the log-rank test. We performed receiver operating characteristic (ROC) curve analysis to evaluate the predictive power of the selected gene signature. mRNA expression values for *CYSLTR1* and *CYSLTR2* derived from the transcriptome datasets were used to build an overall survival classifier (OSC) using Cox proportional hazard regression. The risk scores derived from the five-gene OSC Cox model were used to plot the area under the curve (AUC). The risk scores were calculated using the formula derived from the Cox model. To evaluate the association of gene expression and methylation status in CRC samples with OS, univariate and multivariate Cox proportional hazard regression models were applied, and hazard ratios (HRs) together with 95% confidence intervals (CIs) were calculated to determine the risk of death or cancer recurrence. The multivariate model was adjusted for established prognostic factors such as age, sex, lymph node metastasis (LNM) tumor-node-metastasis (TNM) stage, and tumor size. All patients with incomplete or missing clinical information were excluded from the analysis. To plot the Kaplan–Meier curves, we dichotomized the patients into low- and high-risk groups based on Youden index-derived cutoff values (X-tile software 3.6.1, Rimm Lab, Yale School of Medicine, New Haven, CT, USA). The differences in mRNA levels between normal, tumor and metastasis samples from CRC patients were assessed using a t-test for paired and unpaired data. We performed ROC curve analysis to evaluate the predicted values for lymph node and distant metastasis. M-values for all four CpG sites were used to build a signature for the lymph node and distant metastasis group classifier using a logistic regression model. The risk scores derived from the four-CpG-probe M-values and a logistic model were used to plot the AUCs. Venn diagrams for significant DEGs and heatmaps were generated using the “VennDiagram” and “Plotly” packages, respectively.

## 5. Conclusions

In conclusion, this study first elucidates the oncogenic role of hypomethylation- and hypermethylation-mediated regulation of *CYSLTR1* and *CYSLTR2* expression in CRC, respectively. Moreover, our discovery of *CYSLTR1* and *CYSLTR2* as novel prognostic, lymph node and distant metastasis predictive markers provides important evidence for the clinical significance of the expression and methylation profile of these two CysLTRs in patients with CRC. Further validation of these results in multicenter CRC cohorts could lead to the development of affordable, noninvasive prognostic and predictive markers and population screening assays for CRC patients.

## Figures and Tables

**Table 1:** Distribution of clinical and pathological covariates of in silico and clinical datasets. N—matched normal; AD—adenoma; PT—primary tumor; ME—distant metastasis; CC—Colon cancer; FF—Fresh frozen; FFPE—Formalin fixed paraffin embedded.

Datasets	Sample Type	Tissue Types	Data Types	Platform	Age (Mean ± SD)	Gender			Anatomical Location			TNM Stage			
						Male (n)	Female (n)	Missing (n)	Left (n)	Right (n)	Missing (n)	Stage I	Stage II	Stage III	Stage IV
TCGA COADREAD cohort	N (n = 41)	FF	Tissue mRNA and DNA methylation	RNA-seq and HumanMethylation450k	63.64 ± 13.83	22	19	-	20	16	5	-	-	-	-
	PT (n = 375)				63.56 ± 13.92	207	168	-	187	163	25	57	143	123	52
*GSE77955* *cohort*	N (n = 13)	FF	Tissue mRNA and DNA methylation	Gene expression Microarray and HuanMethylation450k	52.46 ± 10.64	5	5	2	5	5	3	-	-	-	-
	PT (n = 17)				64.55 ± 11.38	11	6	-	11	6	-	-	-	-	17
	AD (n = 17)														
					53.26 ± 8.53	7	8	2	5	7	5	-	-	-	-
	ME (Liver, n = 10 Ovary, n = 1)				66.28 ± 11.24	6	5	-	-	-	-	-	-	-	-
E-MTAB-8148 cohort	N (n = 32)	FF	Tissue mRNA and DNA methylation	RNA-seq and HumanMethylation450k	68.92 ± 16.55	6	26		Missing			Missing			
	PT (n = 216)				66.24 ± 13.10	100	116								
Malmö-CC cohort	N (n = 20)	FFPE	Tissue mRNA and DNA methylation	qRT-PCR and quantitative methylation-specific PCR	65.73 ± 12.73	14	6	-	9	8	3	-	-	-	-
	PT (n = 20)				65.73 ± 12.73	14	6	-	9	8	3	-	5	13	2

## Data Availability

The datasets used and/or analyzed during the current study are available from the corresponding author on request.

## Supplementary Materials

The following supporting information can be downloaded at: https://www.mdpi.com/article/10.3390/ijms24043409/s1.
