# Supplementary material for: DNA Methylation and Gene Expression of the Cysteinyl Leukotriene Receptors as a Prognostic and Metastatic Factor for Colorectal Cancer Patients
Source: Int J Mol Sci. 2023 Feb 8;24(4):3409. doi: 10.3390/ijms24043409 (PMC9963074; doi:10.3390/ijms24043409)
Supplement: Supplementary file 1 [file ijms-24-03409-s001.zip › Supplementary Figures-19-01-2022.docx]

**TCGA-COAD**


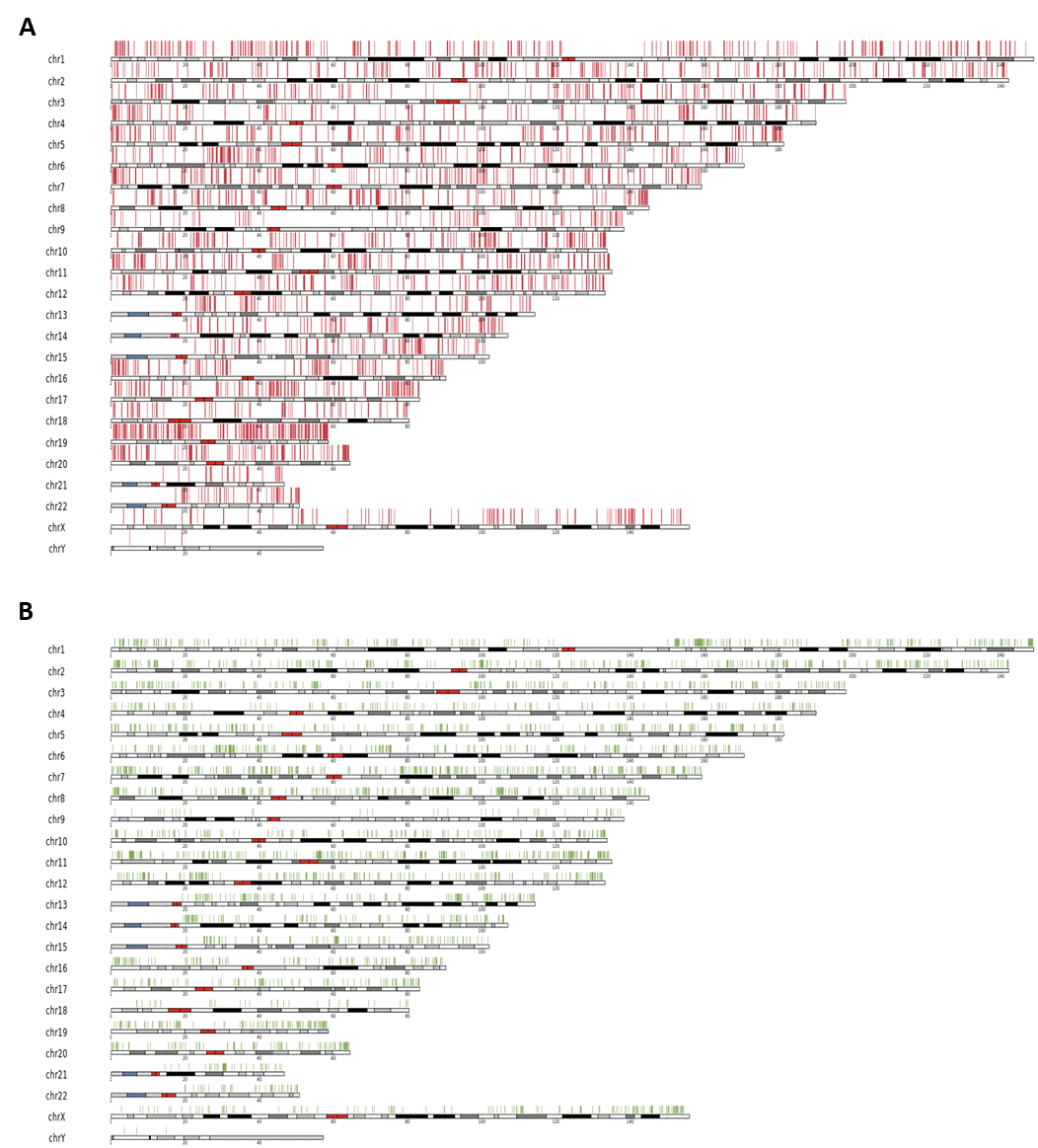


**Supplementary Figure 1.** The global methylated of CpGs in the TCGA-COAD cohort with δ|β| > 0.25 and adjusted P  < 0.05. **(A)** There are 16 122 hyper-methylated CpGs in COAD, with chromosomes 1 to 20 containing the highest number of hyper-methylated CpGs, and chromosome Y containing the least. **(B)** There are 8 736 hypomethylated CpGs in COAD.


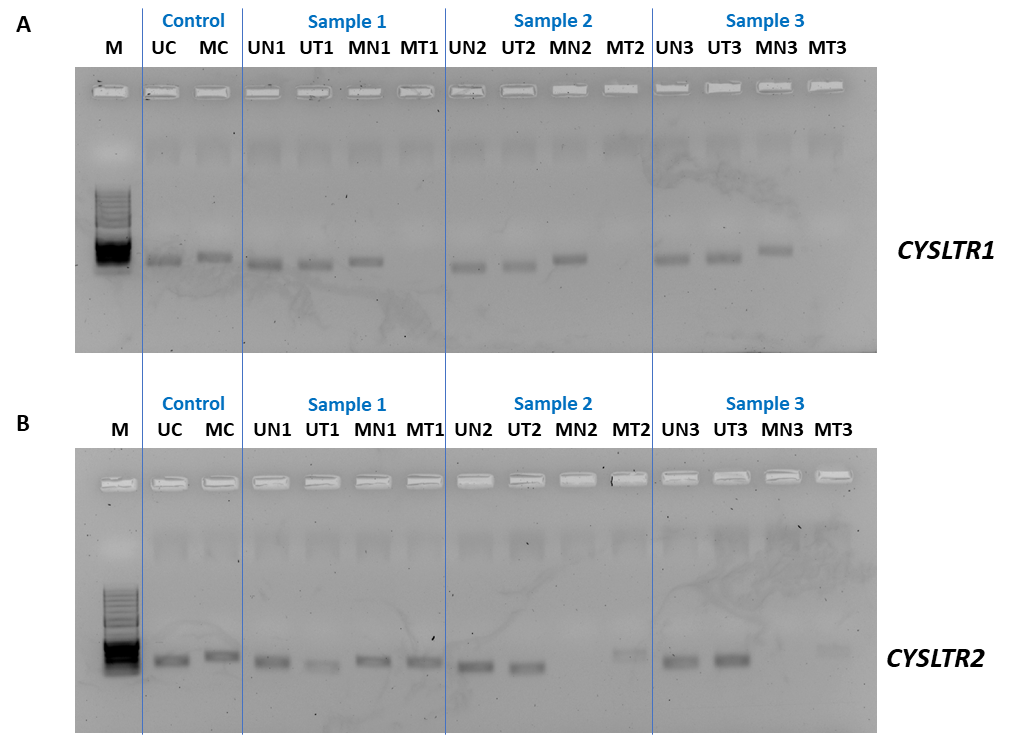


**Supplementary Figure 2.** Agarose gel electrophoresis of methylation-specific PCR (MSP) amplified unmethylated or methylated *CYSLTR1* (A) and *CYSLTR2* (B) genes in three representatives matched normal and tumor samples. M – 100 bp marker, UC – unmethylated control, MC – methylated control, UN – matched normal samples amplified using unmethylated primers, UT - tumor samples amplified using unmethylated primers, MN – matched normal samples amplified using methylated primers, MT - tumor samples amplified using methylated primers.


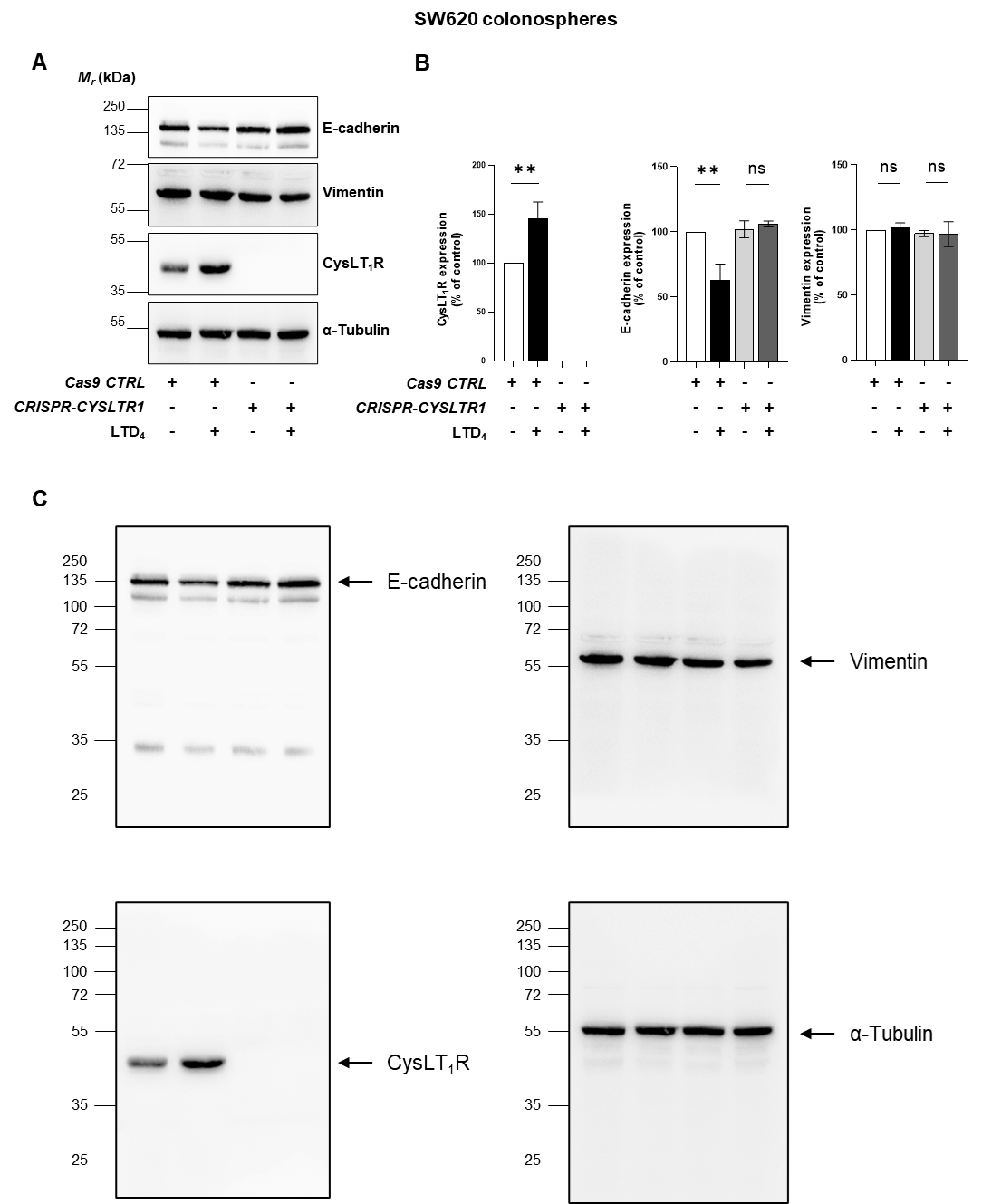


**Supplementary Figure 3.** Protein **e**xpression of the EMT markers E-cadherin and Vimentin in SW620 colonospheres. (A) Representative western blots of E-cadherin, Vimentin proteins in *CRISPR*-based *CYSLTR1* knockdown SW620 colonospheres. (B) Densitometry analysis of CysLT1R, E-cadherin, and Vimentin proteins (% of control). (C) Raw western blots for the E-cadherin, Vimentin, CysLT1R, and α-Tubulin proteins in *Cas9-CTRL* and *CRISPR-CYSLTR1* knockdown samples, treated with or without LTD_4_ in SW620 CC derived colonospheres model. ** P < 0.01; ns = non-significant.

**TCGA-COAD**

# A B


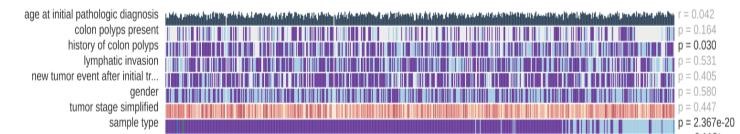

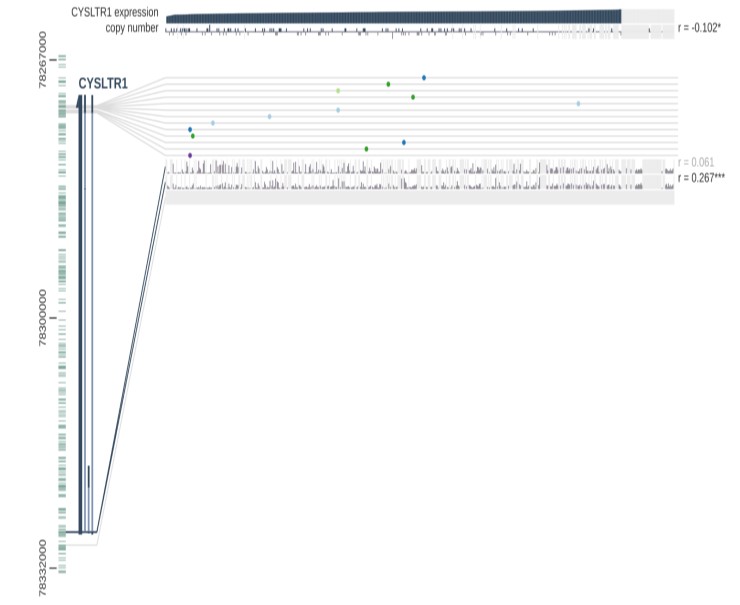

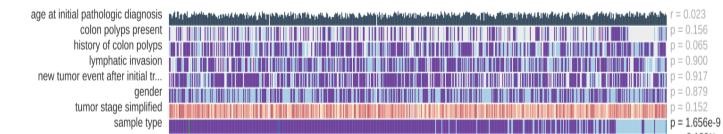

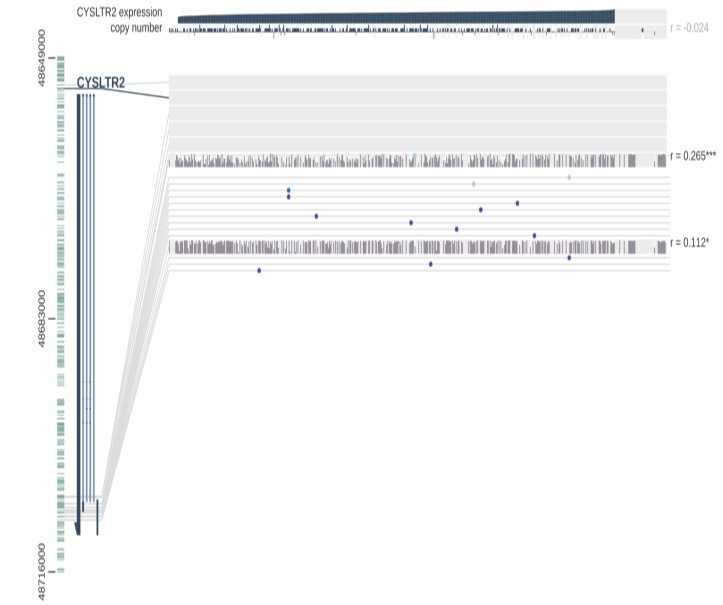


**TCGA-READ**

# D


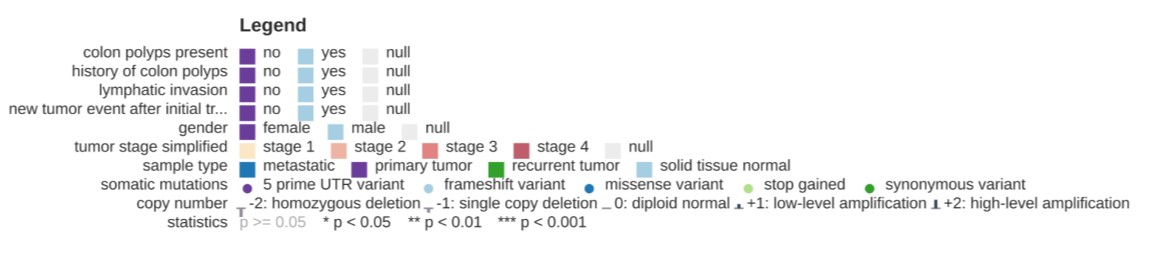

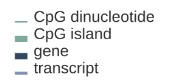

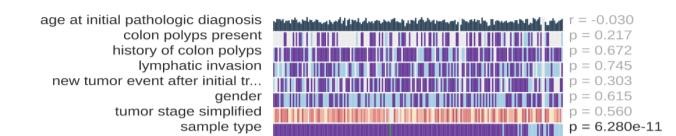

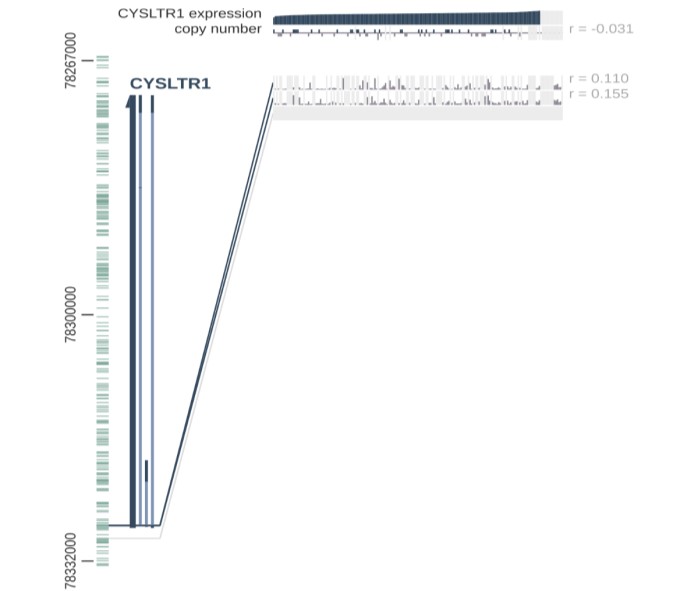

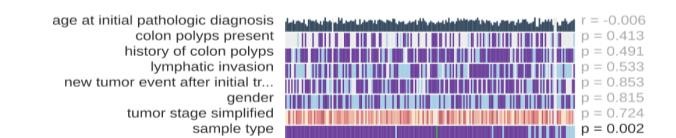

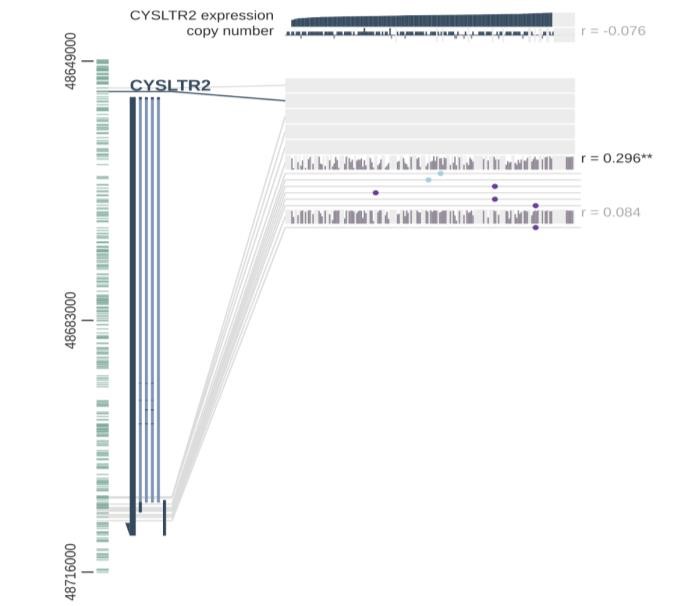


**Supplementary Figure 4.** Association of clinical factors, CpG probe methylation, somatic mutations, and copy number of the *CYSLTR1* and *CYSLTR2* gene expression in TCGA colon (A) and rectum (B) databases. * P < 0.05; ** P < 0.01; *** P < 0.001.
