# Supplementary material for: DNA Methylation and Gene Expression of the Cysteinyl Leukotriene Receptors as a Prognostic and Metastatic Factor for Colorectal Cancer Patients
Source: Int J Mol Sci. 2023 Feb 8;24(4):3409. doi: 10.3390/ijms24043409 (PMC9963074; doi:10.3390/ijms24043409)
Supplement: Supplementary file 1 [file ijms-24-03409-s001.zip › Supplementary Table 2-19-01-2022.docx]

**Supplementary Table 2.** Methylation-specific primers for *CYSLTR1*, *CYSLTR2* and gene-expression primers for *CDH1*, *VIM* and *GAPDH*.

| **Genes** | **Condition** | **Primer’s name** | **Primers sequences (5'-3')** |
| --- | --- | --- | --- |
| ***CYSLTR1*** | Methylated | CYSLTR1-MF | GAAGTAAGTTTTAAGTTTTTAGTAAATTCG |
|  |  | CYSLTR1-MR | ACTCTATAAATAAACTATACTTTTACGACC |
|  | Unmethylated | CYSLTR1-UF | TATTAGTTTTTGTAATTAATTTTTGTTGGT |
|  |  | CYSLTR1-UR | CTATAAATAAACTATACTTTTACAACCCTA |
| ***CYSLTR2*** | Methylated | CYSLTR2-MF | ATTATTATTTTAGAGGTTTTAATTGGATA |
|  |  | CYSLTR2-MR | CTCCTAATACTAATAACATACAAAAACCGA |
|  | Unmethylated | CYSLTR2-UF | ATTATTATTTTAGAGGTTTTAATTGGATA |
|  |  | CYSLTR2-UR | TCCTAATACTAATAACATACAAAAACCAAA |
| ***CDH1*** | Furrowed | CDH1-F | GCCTCCTGAAAAGAGAGTGGAAG |
|  | Reverse | CDH1-R | TGGCAGTGTCTCTCCAAATCCG |
| ***VIM*** | Furrowed | VIM-F | AGGCAAAGCAGGAGTCCACTGA |
|  | Reverse | VIM-R | ATCTGGCGTTCCAGGGACTCAT |
| ***GAPDH*** | Furrowed | GAPDH-F | CGACAACGAATATGGCTA |
|  | Reverse | GAPDH-R | CTGGGAAGGAAAGAAGGG |
